# Identification of Cancer-Associated Proteins in Colorectal Cancer Using Mass Spectrometry

**DOI:** 10.3390/proteomes13030038

**Published:** 2025-08-12

**Authors:** Naoyuki Toyota, Ryo Konno, Shuhei Iwata, Shin Fujita, Yoshio Kodera, Rei Noguchi, Tadashi Kondo, Yusuke Kawashima, Yuki Yoshimatsu

**Affiliations:** 1 Department of Colorectal Surgery, Tochigi Cancer Center, 4-9-13 Yohnan, Utsunomiya 320-0834, Tochigi, Japan; 2 Department of Applied Genomics, Kazusa DNA Research Institute, 2-6-7 Kazusa-kamatari, Kisarazu 292-0818, Chiba, Japan; 3 Division of Rare Cancer Research, National Cancer Center Research Institute, 5-1-1 Tsukiji, Chuo-ku, Tokyo 104-0045, Tsukiji, Japanrenoguch@ncc.go.jp (R.N.); takondo@ncc.go.jp (T.K.); 4 Department of Physics and Center for Disease Proteomics, School of Science, Kitasato University, 1-15-1 Kitasato, Minami-ku, Sagamihara 252-0373, Kanagawa, Japan; kodera@kitasato-u.ac.jp; 5 Department of Cancer Proteogenomics, Tochigi Cancer Center, Tochigi Cancer Center Research Institute, 4-9-13 Yohnan, Utsunomiya 320-0834, Tochigi, Japan; 6 Department of Patient-Derived Cancer Model, Tochigi Cancer Center Research Institute, 4-9-13 Yohnan, Utsunomiya 320-0834, Tochigi, Japan

**Keywords:** colorectal cancer, proteomics, mass spectrometry, protein abundance, biomarker discovery, tumor progression

## Abstract

Background: Colorectal cancer (CRC) is a leading cause of cancer-related mortality worldwide, with a multifactorial etiology involving genetic and environmental factors. Advanced proteomics offers valuable insights into the molecular mechanisms of cancer, identifying proteins that function as mediators in tumor biology. Methods: In this study, we used mass spectrometry-based data-independent acquisition (DIA) to analyze the proteomic landscape of CRC. We compared protein abundance in normal and tumor tissues from 16 patients with CRC to identify cancer-associated proteins and examine their roles in disease progression. Results: The analysis identified 10,329 proteins, including 531 cancer-associated proteins from the Catalogue Of Somatic Mutations In Cancer (COSMIC) database, and 48 proteins specifically linked to CRC. Notably, clusters of proteins showed consistent increases or decreases in abundance across disease stages, suggesting their roles in tumorigenesis and progression. Conclusions: Our findings suggest that proteome abundance trends may contribute to the identification of biomarker candidates and therapeutic targets in colorectal cancer. However, given the limited sample size and lack of subtype stratification, further studies using larger, statistically powered cohorts are warranted to establish clinical relevance. These proteins may provide insights into drug resistance and tumor heterogeneity. Limitations of the study include the inability to detect low-abundance proteins and reliance on protein abundance rather than functional activity. Future complementary approaches, such as affinity proteomics, are suggested to address these limitations.

## 1. Introduction

Colorectal cancer (CRC) remains one of the most common and lethal cancers worldwide, contributing to significant morbidity and mortality across diverse populations. Globally, it is the third most commonly diagnosed cancer in men and the second in women, with approximately 1.9 million new cases and nearly 935,000 deaths annually [1,2]. This growing burden poses a major challenge to healthcare systems, particularly in regions with limited access to advanced diagnostic tools and effective treatments. The complexity of CRC stems from its multifactorial etiology, in which both genetic predispositions and environmental factors play crucial roles [3,4]. Risk factors such as diet, smoking, alcohol consumption, and chronic inflammation contribute to the progression of normal epithelial cells to adenocarcinomas, accompanied by various molecular alterations including genetic mutations, epigenetic changes, and chromosomal instability [5]. As a result, there is an urgent need for more precise molecular characterization of CRC to facilitate early diagnosis [6], monitor disease progression [7], and identify novel therapeutic targets [8].

The advent of proteomics—the large-scale study of proteins, their structures, and functions—has revolutionized cancer research by providing deeper insights into the molecular mechanisms underlying tumor progression [9,10,11]. While genomic and transcriptomic analyses are essential for identifying mutations and transcriptional changes, proteomics enables direct assessment of the active functional components of the cell. Proteins are critical to nearly every biological process, including cell signaling, metabolism, apoptosis, and cell division. Alterations in the proteome often provide crucial insights into tumor biology. Indeed, many studies have shown poor correlation between mRNA and protein abundance levels, reinforcing the importance of direct protein measurement [12,13,14,15,16,17,18,19,20,21,22,23,24]. DNA and RNA are relatively stable molecules in terms of their structure, as they are not extensively post-replicatively or post-transcriptionally modified. However, RNA itself is highly prone to degradation, requiring careful handling during transcriptomic analyses. In contrast, proteins undergo dynamic post-translational modifications—such as phosphorylation or ubiquitination—that directly influence their function, localization, and interactions [25,26]. Thus, proteomic profiling offers a more comprehensive and functionally relevant perspective on disease states.

In this study, we utilized mass spectrometry-based proteomics to compare protein abundance profiles in matched tumor and normal tissues from colorectal cancer patients. Mass spectrometry is one of the most powerful tools available for proteomic analysis, enabling high-throughput and highly sensitive identification and quantification of thousands of proteins [27,28,29]. However, many cancer-related proteins are expressed at low abundance and are difficult to detect using conventional data-dependent acquisition (DDA) methods. To address this, we employed data-independent acquisition (DIA), a mass spectrometry approach that improves reproducibility and detection depth in proteomic studies. DIA-based LC-MS/MS is a peptide-centric, label-free, quantitative proteogenomic technique that enables comprehensive protein profiling. While DIA has gained momentum in recent years, its application to diverse clinical CRC samples remains limited.

Recent studies using DIA-MS have made substantial progress in subclassifying CRC and linking proteomic features to clinical outcomes. For example, three proteomic subtypes (P1–P3) have been identified, with the P3 subtype associated with poor prognosis, immune evasion, and enhanced metastatic potential [30]. Additionally, integrated serum proteomic and metabolomic analyses have revealed metabolic reprogramming and trace element associations with CRC pathophysiology [31,32]. These findings emphasize the growing translational potential of DIA-MS and the importance of subtype-specific molecular characterization.

Building upon this context, our study aims to contribute complementary insights by analyzing consistent protein abundance changes across CRC clinical stages in a smaller, well-defined cohort. By comparing matched tumors and adjacent normal tissues, we provide an orthogonal view of the CRC proteome across disease progression. This foundational dataset may inform future efforts in biomarker discovery and subtype-specific classification.

## 2. Materials and Methods

### 2.1. Surgical Specimens

Tumor tissues were obtained from the Tochigi Cancer Biobank. The samples were collected from surgical specimens under visual inspection and guidance from a certified pathologist. They were then stored in vapor-phase liquid nitrogen until further use. Informed consent was obtained from all donors. The use of biobank-derived tumor tissues, along with relevant clinical and pathological data, for this study was approved by the Ethical Committee of the Tochigi Cancer Center (Approval No. 21-A037). This study included tumor tissues and their matched adjacent normal counterparts from 16 patients with CRC. A summary of the donors’ clinical and pathological information is provided in Table S1.

### 2.2. Protein Extraction and Digestion

Tissue samples were dissolved in 200 µL of 100 mM Tris-HCl (pH 8.5) containing 4% sodium dodecyl sulfate (SDS, Fujifilm Wako Pure Chemical Corp., Osaka, Japan) and sonicated using a Bioruptor II equipment (SONIC Bio Co., Saitama, Japan) set at high intensity and using a “30 s On/Off” cycle for 30 min. After centrifugation at 20,000× *g* for 15 min at 4 °C, the supernatant was transferred to a fresh tube. Protein abundance was measured using a NanoDrop spectrophotometer (Thermo Fisher Scientific, Waltham, MA, USA), and the concentration was adjusted to 0.5 µg/µL with 100 mM Tris-HCl (pH 8.5) containing 4% SDS. Reduction took place by adding 2 µL of 200 mM Bond-Breaker TCEP solution (Thermo Fisher Scientific) to 40 μL of the protein extracts and incubating them for 10 min at 80 °C. Alkylation of reduced cysteine residues was achieved by adding 2 µL of 375 mM iodoacetamide and 200 mM TEAB, and incubating the samples during 30 min in the dark at room temperature. The samples were then subjected to clean up and digestion with single-pot solid phase-enhanced sample preparation (SP3), with minor modifications from the previously reported protocol [33,34], Briefly, magnetic beads (20 µL) were added to the alkylated sample followed by ethel alcohol to bring the final concentration to 75% (*v*/*v*). The samples were mixed for 10 min. The supernatant was discarded, and the pellet was washed with 800 µL of 80% ethyl alcohol twice. The beads were resuspended in 100 µL of 50 mM Tris-HCl (pH 8.0) containing 200 ng of trypsin (Promega) and of lysylendopeptidase (Fujifilm Wako Pure Chemical Corp.), and mixed gently for 18 h at 37 °C. The digested sample was acidified with 20 μL of 5% trifluoroacetic acid (TFA) and mixed using a vortex for a few minutes, followed by a spin down. The supernatant was desalted using MonoSpin C18 (GL Science, Tokyo, Japan) according to the manufacturer’s protocol. Peptides were eluted using 50% acetonitrile (ACN) and 0.1% TFA, and then freeze-dried. Freeze-dried samples were resuspended in 20 µL of 3% ACN and 0.1% formic acid using a combination of vortexing and ultrasonic agitation (30 s on/30 s off, at high intensity) during 10 min at 4 °C.

### 2.3. Liquid Chromatography–Mass Spectrometry/Mass Spectrometry (LC-MS/MS)

Peptides were directly loaded onto a 75 μm × 30 cm nanoLC column (CoAnn Technologies, Richland, WA, USA) at 50 °C and then separated using a 100 min gradient with solvent A (0.1% formic acid [FA] in water) and solvent B (0.1% FA in 80% acetonitrile [ACN]). The gradient conditions were as follows: 0 min, 5% B; 86 min, 33% B; 92 min, 70% B; and 100 min, 70% B. The flow rate was set to 150 nL/min using the UltiMate 3000 RSLCnano LC system (Thermo Fisher Scientific). Peptides eluted from the column were analyzed on an Orbitrap Exploris 480 Mass Spectrometer (Thermo Fisher Scientific) using the InSpIon system [35]. MS1 spectra were collected in the *m*/*z* range of 495–865 at a resolution of 15,000, with an automatic gain control (AGC) target of 3 × 106 ions and a maximum injection time of “auto”. MS2 spectra were acquired in the *m*/*z* range of 200–1800 at a resolution of 45,000, with an AGC target of 3 × 106 ions, a maximum injection time of “auto”, and a stepped normalized collision energy of 26%. The isolation width for MS2 was set to 6 Th, and an optimized window arrangement for the *m*/*z* 500–860 range was applied in Xcalibur 4.4 (Thermo Fisher Scientific). Each sample was analyzed in a single technical replicate.

### 2.4. Protein Identification

Raw data files were searched against an in silico human spectral library using DIA-NN (version 1.8.1; https://github.com/vdemichev/DiaNN; accessed on 1 February 2022) [36]. An in silico spectral library was first generated from the human protein sequence database (proteome ID UP000005640, 20,591 entries, downloaded in March 2023) using the DIA-NN. The parameters for spectral library generation included trypsin as the digestion enzyme, with allowance for one missed cleavage, peptide length ranging from 7 to 45 amino acids, precursor charges from 2 to 4, precursor *m*/*z* from 495 to 865, and fragment ion *m*/*z* values from 200 to 1800. Additionally, the following options were enabled: “FASTA digest for library-free search/library generation”, “deep learning-based spectra, retention times, and ion mobility prediction”, “N-terminal methionine (M) excision”, and “C-terminal carbamidomethylation”. The search parameters for DIA-NN, using the human protein sequence database and the generated spectral library, were as follows: MS1 accuracy of 10 ppm; MS2 accuracy of 10 ppm; and a static modification of cysteine carbamidomethylation. Additionally, the following options were enabled: “Remove likely interferences”, “Use isotopologues”, “Unrelated runs”, and “Match between run”. The DIA-NN command line also included the peak translation and relaxed-prof-inf commands. The protein identification threshold was set at <1% for both peptide and protein false discovery rates (FDRs).

### 2.5. Statistical Analyses

In the two-group analysis of healthy and diseased samples, protein abundance levels were log2-transformed. Proteins were selected based on valid values detected in at least 70% of the samples within at least one experimental group. Missing values were imputed by drawing random numbers from a normal distribution (downshift = 2.4; width = 0.3) using Perseus v1.6.15.0 [35]. Perseus v1.6.15.0 was used for principal component analysis (PCA) and Pearson’s correlation coefficient heatmap analysis with hierarchical clustering. Welch’s t-test was performed to compare non-tumor and tumor tissues, identifying significant proteins with a *p*-value < 0.05 and a fold change > 2 or <0.5. For each clinical stage, proteins were selected based on valid values detected in at least 70% of the samples in at least one experimental group, following the Log2 transformation of protein intensities applied in the two-group analysis. The missing values were imputed using random numbers drawn from a normal distribution (downshift = 1.8, width = 0.3). One-way ANOVA was conducted for each clinical stage, with multiple testing corrections using the permutation-based FDR function in the Perseus software. Proteins with a significant FDR (<0.01) were used for subsequent analyses. For the hierarchical cluster analysis, the median value for each group was calculated and converted to a normalized z-score. Default parameters were applied, with column clustering disabled and samples ordered by clinical stage. The resulting clusters were divided into 30 groups based on the top hierarchy. Heatmaps and profile plots for each cluster were generated using Python (version 3.9.13, 2022) with the Seaborn (version 0.13.2, 2024) and Matplotlib (version 3.7.2, 2023) libraries.

## 3. Results

### 3.1. Overall Features of the Proteome of Non-Tumor and Tumor Tissues

**Figure 1:**
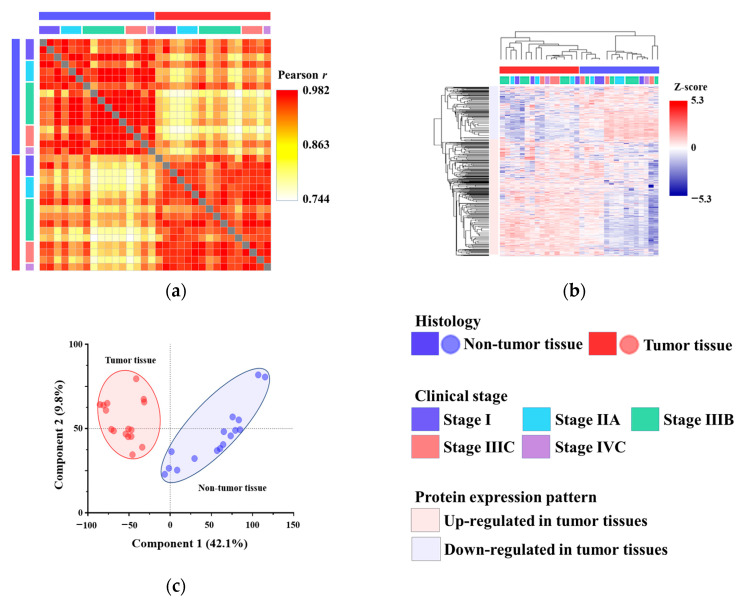
Overview of proteome features in colorectal cancer. (**a**) Correlation matrix of non-tumor and tumor tissues. Tissues with identical histological characteristics exhibited similar abundance patterns. (**b**) Unsupervised hierarchical clustering of tumor and non-tumor tissues based on protein abundance profiles. Tissues were grouped according to their histological diagnosis; however, the clinical stage was not reflected in the classification. (**c**) Principal component analysis (PCA) of non-tumor and tumor tissues based on protein abundance profiles. Tissues were positioned in a two-dimensional space, clearly separating tumor and non-tumor tissues according to their histology.

Proteins extracted from tumor tissues were digested with trypsin and analyzed via mass spectrometry. In total, 10,329 proteins were identified across all samples, covering the majority of proteins abundant in both tumor and non-tumor tissues. The detected proteins are summarized in Table S2. We assessed the overall characteristics of the identified proteins to determine whether their abundance patterns aligned with histological observations, as well as clinical and pathological data. We found that protein samples within the same histological category, such as tumor and non-tumor tissues, exhibited high similarity, as demonstrated by the correlation matrix (Figure 1a). The numerical data for the correlation matrix are provided in Table S3. Next, hierarchical clustering of the protein abundance data was used to group the samples according to their histological classifications, such as tumor and non-tumor tissues (Figure 1b). Notably, tumor tissues were not grouped according to clinical stage. The proteins were classified into two groups: those with decreased abundance and those with increased abundance in tumor tissues compared to non-tumor tissues (Figure 1b). Additionally, PCA revealed that the protein samples clustered according to their histological classifications (Figure 1c).

### 3.2. Proteins with Differential Abundance Between Non-Tumor and Tumor Tissues

We captured the overall features of proteins with differential abundance between tumor and non-tumor tissues. We selected proteins whose abundance levels differed between tumor and non-tumor tissues by at least 2-fold, with statistical significance defined as a *p*-value less than 0.05. A total of 2642 proteins were selected; 1475 proteins were increased in abundance, and 1167 proteins were decreased in abundance. The data are summarized in Table S4.

**Figure 2:**
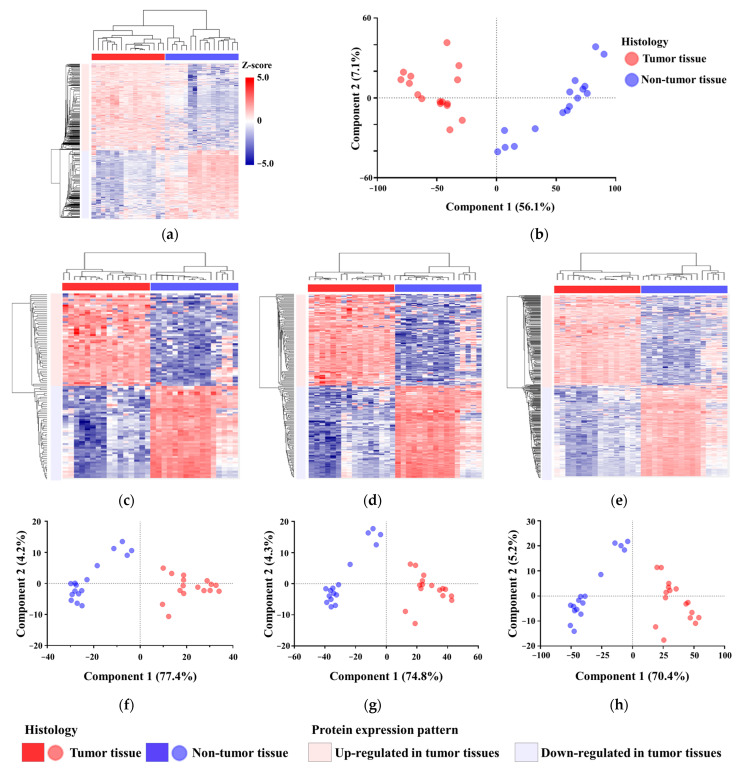
Overall features of proteins with differential abundance. (**a**) Abundance profiles of proteins with differential abundance (>2-fold difference, *p* < 0.05) between tumor and non-tumor tissues were used to characterize colorectal cancer tissues. Unsupervised hierarchical clustering revealed that tissues with the same histological appearance exhibited similar abundance patterns. (**b**) PCA of non-tumor and tumor tissues based on protein abundance profiles. Dimensionality reduction positioned samples in a two-dimensional space, distinctly separating tissues according to their histology. (**c**–**e**) Unsupervised hierarchical clustering based on the abundance data of the top 50 (**c**), top 100 (**d**), and top 200 (**e**) proteins with increased and decreased abundance, respectively. (**f**–**h**) PCA using the abundance data of the top 50 (**f**), top 100 (**g**), and top 200 (**h**) proteins. PCA, principal component analysis.

Protein samples were classified based on the abundance patterns of differentially abundant proteins (Figure 2a). Although hierarchical clustering generated decisive results, the protein samples did not perfectly reflect the histology of the original tissues in the cluster tree. However, visual inspection of the heatmap revealed that the samples could be categorized into two groups, which were equivalent to those of the histological classification (Figure 2a). In PCA, the protein samples were separated, reflecting the histology of the original tissues (Figure 2b). We ranked the proteins based on their abundance differences between tumor and non-tumor tissues. The abundance data for the top 50 (Table S5), 100 (Table S6), and 200 proteins (Table S7) with increased and decreased abundance were analyzed using hierarchical clustering (Figure 2c–e) and PCA (Figure 2f–h). Using extensively selected proteins, the protein samples were divided into tumor and non-tumor groups.

### 3.3. Cancer-Associated Proteins Observed in This Study

We also explored the number of cancer-associated proteins identified in this study. They were selected based on the Catalogue of Somatic Mutations in Cancer (COSMIC, https://www.cosmickb.org/about/ (accessed on 9 September 2024)). The COSMIC defines 748 proteins as cancer-associated proteins based on the recurrent mutations observed in malignancies (Table S8). Of these, 531 proteins were identified (Table S9). Additionally, COSMIC includes 65 colorectal cancer-associated proteins (Table S10), of which 48 were observed in our study (Table S11). These findings indicate that our analysis captured 71% of all cancer-associated proteins (531/748) and 75% of the colorectal cancer-associated proteins (48/64). However, 217 cancer-associated proteins recorded in the COSMIC database were not detected in our study (Table S12). This group included 16 proteins with recurrent mutations in colorectal cancer (Table S13). Importantly, all 48 colorectal cancer-associated proteins identified in this study were consistently detected across all examined tissue samples.

### 3.4. Proteins Associated with Disease Progression

**Figure 3:**
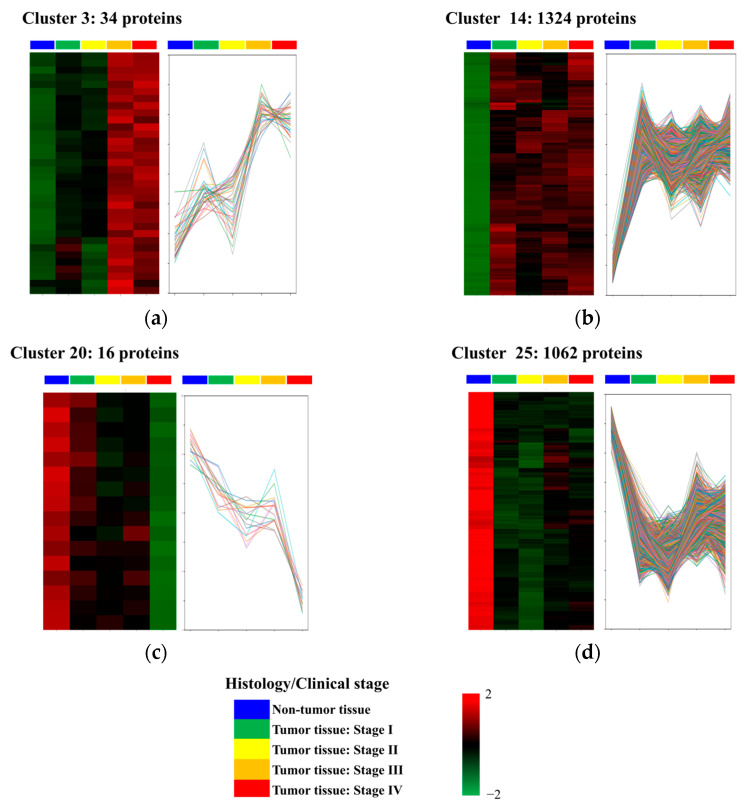
Patterns of differentially abundant proteins. (**a**,**b**) Clusters of proteins whose abundance levels increased with disease progression across colorectal cancer stages. Cluster 3 contains 34 proteins, and Cluster 14 contains 1324 proteins. These proteins show higher abundance in tumor tissues at Stage I compared to non-tumor tissues and remain elevated through Stages II to IV. (**c**,**d**) Clusters of proteins whose abundance levels decreased with disease progression. Cluster 20 includes 16 proteins, and Cluster 25 includes 1062 proteins. These proteins show lower abundance in tumor tissues at Stage I compared to non-tumor tissues and remain decreased through Stages II to IV. Heatmaps depict protein abundance levels, while line graphs illustrate abundance trends across clinical stages. Histology/clinical stages are represented as follows: blue (non-tumor tissue), green (Stage I), yellow (Stage II), orange (Stage III), and red (Stage IV).

We focused on analyzing trends in protein abundance levels with disease progression. The two types of proteins were selected based on their abundance patterns. Through cluster analysis, the proteins in this study were categorized into 30 clusters according to their abundance profiles in the tissue samples (Figure S1 and Table S18). First, we identified proteins whose abundance levels were more increased in tumor tissues at stage I than in non-tumor tissues and remained increased in abundance as the disease progressed from stage I to stage IV. Proteins in clusters 3 and 14 were categorized into this group (Figure 3a,b). Clusters 3 and 14 included 34 and 1324 proteins, respectively (Tables S14 and S15). Second, we selected proteins whose abundance levels were lower in tumor tissues at stage I compared to non-tumor tissues, and which remained decreased throughout disease progression from stage I to stage IV. The proteins in clusters 16 and 25 exhibited these characteristics (Figure 3c,d). Clusters 16 and 25 contained 16 and 1062 proteins, respectively (Tables S16 and S17).

## 4. Discussion

The significance of proteomic studies in comparing non-tumor and tumor tissues lies in their ability to provide detailed insights into the molecular aberrations that drive cancer progression. By analyzing the complete set of proteins, including those differentially abundant between tumor and non-tumor tissues, we can identify potential candidates for clinical applications, such as biomarkers or therapeutic targets. For example, proteins with differential abundance between tumor and non-tumor tissues may serve as biomarkers for diagnosis or disease monitoring, enabling the early detection of cancer and more accurate prediction of treatment outcomes. Proteins whose abundance levels are associated with clinically important features, such as metastasis and treatment resistance, provide new hypotheses for cancer progression. These hypotheses can lead to a deeper understanding of signaling pathways, metabolic regulation, and immune responses. However, compared to previous studies that identified clinically relevant CRC subtypes through proteomic clustering [1,2,3], our work did not include explicit subtype classification or survival analysis due to the limited cohort size. Our findings are exploratory and provide a foundational proteomic dataset focusing on early-to-late-stage comparisons, rather than clinical stratification or prognostic modeling. We acknowledge that the absence of such analysis may limit the interpretability of our data in terms of translational utility. Nevertheless, proteins that consistently changed across stages could serve as preliminary candidates for future subtype-associated biomarker discovery when integrated with larger datasets and clinical annotations. Additionally, the increased abundance of proliferation-regulatory proteins in tumor tissues presents a potential therapeutic target. Therefore, the identification of proteins with unique abundance patterns in tumor tissues is an initial step toward various biological studies.

Mass spectrometry (MS)-based proteomics has revolutionized cancer research by providing deep insights into the proteomic landscape of cancer cells, allowing the identification of biomarkers, therapeutic targets, and molecular mechanisms [36]. Despite these advantages, this technique has several limitations. One of the major challenges of MS-based proteomics is the detection of low-abundance proteins, which is critical for cancer research. Many cancer-related proteins, such as tumor suppressors, transcription factors, and signaling proteins, are abundant at very low levels, making them difficult to detect among the highly abundant proteins. These low-abundance proteins are often crucial for understanding cancer biology but may be overshadowed by more abundant proteins in complex samples. Furthermore, while mass spectrometry-based proteomics can provide quantitative data, achieving precise quantitation using label-free methods is challenging, especially for low-abundance proteins. This can lead to variability in results, particularly when comparing protein levels across samples or studies.

DIA mass spectrometry provides remarkable advantages over traditional data-dependent acquisition approaches owing to its improved reproducibility, ability to quantify low-abundance proteins, and enhanced sensitivity and consistency across large sample sets [37]. These characteristics make DIA an ideal choice for high-throughput, large-scale proteomic studies. Additionally, the ability to reanalyze post-acquisition data allows researchers to extract more value from a single experiment, making it a cost-effective and powerful tool in modern proteomics. Although DIA has some limitations, such as the need for sophisticated computational tools for data analysis, its advantages make it a leading technique in proteomics.

These findings highlight the potential of proteomics to provide a more comprehensive understanding of colorectal cancer at the molecular level. We identified 10,329 proteins, covering the majority of proteins abundant in tumor and non-tumor tissues. Our results demonstrate the potential of proteomics, not only in the number of observed proteins but also in the diversity of protein categories. In this study, 71% of the cancer-associated proteins were identified, including 75% of those associated with colorectal cancer. These observations underscore the promising application of cancer proteomics. However, 217 cancer-associated proteins, including 16 colorectal cancer-associated proteins, were not observed, highlighting the current limitation of DIA mass spectrometry and the need for complementary approaches to detect non-observable proteins. Affinity proteomics, such as the Proximity Extension Assay [38] and the SomaScan platform [39], have been developed, and these technologies have the potential to detect proteins that are not accessible by DIA mass spectrometry. No single proteomics technology can fully cover the entire proteome. While affinity-based proteomic methods such as SomaScan and Proximity Extension Assay offer high sensitivity for low-abundance proteins, they also present limitations. In particular, these technologies may not distinguish specific proteoforms and are incompatible with harsh extraction buffers (e.g., surfactants and chaotropes) used in LC-MS/MS workflows. Therefore, such assays are typically optimized for plasma or serum samples rather than solid tissues.

The abundance trend along with disease progression is important when interpreting the functional significance of an aberrant protein status. As the number of samples in this study was limited, the increase or decrease in abundance of certain proteins may have been observed only in the examined samples, and this possibility cannot be excluded. With this in mind, we focused on proteins with consistent increases or decreases in abundance in tumor tissues rather than in non-tumor tissues during carcinogenesis and disease progression. Although this study successfully identified many proteins, their functional significance requires further investigation. Proteins with distinct abundance patterns across colorectal cancer stages may serve as potential biomarkers; however, their roles in disease biology must be validated through functional studies. This highlights the importance of integrating abundance data with analyses of protein activity, post-translational modifications, and their interactions, as these factors are essential for understanding biological relevance.

Although we used the most advanced mass spectrometry technology, our approach has fundamental limitations. While we reported proteins with differential abundance levels between tumor and non-tumor tissues, it remains uncertain whether proteins with more prominent abundance differences have greater functional significance in colorectal cancer biology. Protein function does not always correlate with abundance levels; factors such as enzymatic activity, post-translational modifications, and protein–protein interactions should be considered alongside abundance studies. Additionally, the limited number of cases in this study indicates that our findings alone cannot identify definitive biomarkers or therapeutic targets. Data from a larger, statistically significant cohort complemented by functional validation and additional samples are essential for drawing clinically applicable conclusions. Furthermore, our analysis does not account for CRC subtype heterogeneity, which has been shown to influence proteomic profiles and clinical outcomes [30,31,32]. The absence of correlation with immune evasion or metabolic reprogramming pathways, as highlighted in prior literature, represents a key limitation of this work. Future efforts should include subtype-aware design, integration of multi-omics data, and survival analyses to enhance the translational potential of identified protein markers. The supplemental data provided in this study will serve as valuable resources for future research. Moreover, this study did not consider the complexity of the proteome by examining proteoforms—structurally and functionally distinct protein species arising from alternative splicing, post-translational modifications, and genetic variations. Understanding the proteome information is critical for accurately interpreting proteomic data and identifying clinically relevant biomarkers for CRC.

While this study successfully identified a large number of proteins with differential abundance using DIA-based proteomics, we acknowledge that protein abundance alone does not fully reflect protein activity, post-translational modifications (PTMs), or interaction networks—features that are often critical for understanding disease mechanisms and therapeutic vulnerabilities. Current DIA workflows are not yet able to capture these additional layers of information comprehensively.

To further elucidate the biological roles of the identified proteins, future studies should incorporate complementary approaches, such as phosphoproteomics, protein interaction mapping, and activity-based profiling. To address this issue, data-dependent acquisition (DDA)-based workflows remain useful for PTM-specific studies, as they allow for more targeted identification of modified peptides. Additionally, antibody-based proteomic platforms, such as reverse-phase protein arrays (RPPA), can be used to detect specific PTMs with high sensitivity. Additionally, the integration of abundance data with pathway enrichment analyses and curated literature reviews will be essential for deriving mechanistic insights and identifying clinically relevant targets. Such analyses were not performed in the present study due to the limited sample size and scope but are planned for future work involving larger and more diverse clinical datasets.

## Data Availability

The mass spectrometry proteomic data have been deposited in the Proteo-meXchange Consortium via the jPOST partner repository, with dataset identifiers PXD058672 for ProteomeXchange and JPST003422 for jPOST.

## Supplementary Materials

The following supporting information can be downloaded at https://www.mdpi.com/article/10.3390/proteomes13030038/s1, Table S1: Clinical information about donors; Table S2: Protein abundance data; Table S3: Numerical data for correlation matrix; Table S4: Tumor vs. non-tumor abundance 2642 proteins; Table S5: Top 50 proteins; Table S6: Top 100 proteins; Table S7: Top 200 proteins; Table S8: COSMIC 748 cancer-associated proteins; Table S9: COSMIC 531 observed cancer-associated proteins; Table S10: COSMIC 64 colorectal cancer-associated proteins; Table S11: COSMIC 45 observed colorectal cancer-associated proteins; Table S12: COSMIC 217 non-observed cancer-associated proteins; Table S13: COSMIC 16 non-observed colorectal cancer-associated proteins; Table S14: Figure 3a—Cluster 3 (34 proteins); Table S15: Figure 3b—Cluster 14 (1324 proteins); Table S16: Figure 3c—Cluster 20 (16 proteins); Table S17: Figure 3d—Cluster 25 (1062 proteins); Table S18: All Clusters 1–30; Figure S1: Protein abundance profiles across histological stages of colorectal cancer.

## Abbreviations

The following abbreviations are used in this manuscript:

ACN	Acetonitrile
AGC	Automatic gain control
COSMIC	Catalogue of Somatic Mutations in Cancer
CRC	Colorectal cancer
DIA	Data-independent acquisition
FA	Formic acid
FDR	False discovery rate
MS	Mass spectrometry
PCA	Principal component analysis
SDS	Sodium dodecyl sulfate
TFA	Trifluoroacetic acid
