# Supplementary material for: Identification of Cancer-Associated Proteins in Colorectal Cancer Using Mass Spectrometry
Source: Proteomes. 2025 Aug 12;13(3):38. doi: 10.3390/proteomes13030038 (PMC12372073; doi:10.3390/proteomes13030038)
Supplement: Supplementary file 1 [file proteomes-13-00038-s001.zip › Supplementary Figure S1.pptx]

## Slide 1
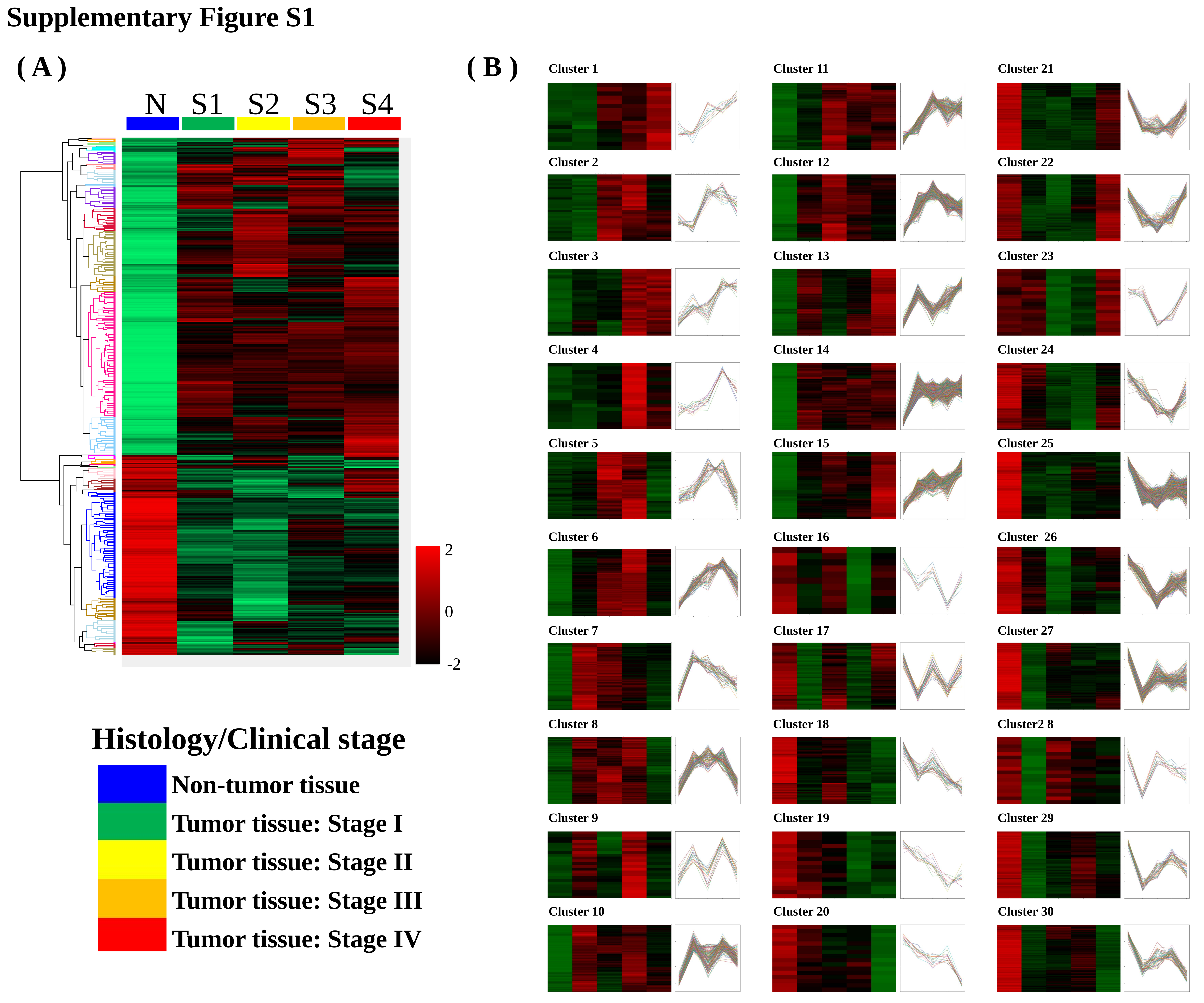

Supplementary Figure S1
( A )
( B )
Cluster 1
Cluster 11
Cluster 21
N
S1
S2
S3
S4
Cluster 2
Cluster 12
Cluster 22
Cluster 3
Cluster 13
Cluster 23
Cluster 4
Cluster 14
Cluster 24
Cluster 5
Cluster 15
Cluster 25
Cluster 6
Cluster 16
Cluster 26
2
0
Cluster 7
Cluster 17
Cluster 27
-2
Cluster 8
Cluster 18
Cluster2 8
Histology/Clinical stage
Non-tumor tissue
Tumor tissue: Stage I
Cluster 9
Cluster 19
Cluster 29
Tumor tissue: Stage II
Tumor tissue: Stage III
Cluster 10
Cluster 20
Cluster 30
Tumor tissue: Stage IV
