# Supplementary material for: Identification of Cancer-Associated Proteins in Colorectal Cancer Using Mass Spectrometry
Source: Proteomes. 2025 Aug 12;13(3):38. doi: 10.3390/proteomes13030038 (PMC12372073; doi:10.3390/proteomes13030038)
Supplement: Supplementary file 1 [file proteomes-13-00038-s001.zip › Supplementary Materials.pdf]

Tables

Supplementary Table S1 Clinical information of donor patients

Supplementary Table S2 Protein expression data

Supplementary Table S3 Numerical data for correlation matrix

Supplementary Table S4 Tumor vs Non-Tumor Expression 2,642 Proteins

Supplementary Table S5 Top50

Supplementary Table S6 Top100

Supplementary Table S7 Top200

Supplementary Table S8 All Clusters 130

Supplementary Table S9 Fig3A_Clustre3_34 protein

Supplementary Table S10 Fig3B_Clustre14_1324 protein

Supplementary Table S11 Fig3C_Clustre20_16 protein

Supplementary Table S12 Fig3D_Clustre25_1062 protein

Supplementary Table S13 COSMIC 748 cancer associated proteins

Supplementary Table S14 COSMIC 531 observed cancer associated proteins

Supplementary Table S15 COSMIC 64 colorectal cancer associated proteins

Supplementary Table S16 COSMIC 48 observed colorectal cancer associated proteins

Supplementary Table S17 COSMIC 217 not-observed cancer associated proteins

Supplementary Table S18 COSMIC 16 not-observed colorectal cancer associated proteins

Figure Legends

Supplementary Figure S1. Protein expression profiles across histological stages of colorectal cancer

(A) Heatmap displaying hierarchical clustering of protein expression profiles across different histological stages of colorectal cancer. Samples are categorized as non-tumor tissue (blue) and tumor tissues at various clinical stages: Stage I (green), Stage II (yellow), Stage III (orange), and Stage IV (red). Rows represent proteins, and columns represent individual samples. Protein expression levels are indicated by a color scale ranging from green (low expression) to red (high expression), with black representing median expression levels. The dendrogram clusters are color-coded from 1 to 30, starting from the top of the heatmap. (B) Heatmap and line plot visualizations for each of the 30 clusters identified in (A). Each heatmap shows the expression

patterns of proteins within a cluster, while the line plots to the right illustrate the average expression trends. These clusters highlight stage-specific patterns of protein expression, revealing systematic variation across non-tumor and tumor tissues at different stages of colorectal cancer.
